# DNA sequencing in the classroom: complete genome sequence of two earwig (Dermaptera; Insecta) species

**DOI:** 10.1186/s40659-023-00414-9

**Published:** 2023-02-17

**Authors:** Sanae Kobayashi, Jonathan E. Maldonado, Alexis Gaete, Ingrid Araya, Constanza Aguado-Norese, Nicolás Cumplido, Sebastián Díaz, Alonso Espinoza, Edelmira Fernández, Felipe Gajardo, Felipe González-Ordenes, Khantati Hauyon, Piedad Maldonado, Rodrigo Maldonado, Isabel Pochet, Aníbal Riveros, Paula Sandoval, Ailynne Sepúlveda-González, Camila Stuardo, Patricio Tapia-Reyes, Carolina Thornton, Soledad Undurraga, Macarena Varas, Camilo Valdivieso, School Earwig Genome Consortium, Alan Phillips, Alejandro Aros, Alexandra Alarcón, Alonso Mendiboure, Alyson Sepúlveda, Amalia Zepeda, Angela Bustamante, Angelo Russu, Anselmo Martínez, Antonia Inostroza, Antonio Palma, Bárbara Ponce, Belén Báez, Belén Dianta, Benjamín Zenteno, Berenice Jelvez, Brisa Henríquez, Camila Concha, Catalina Fuentes, Catalina Morales, Claudia Inostrosa, Claudio Valenzuela, Constanza Dercolto, Cristian Malebrán, Damián González, Daniel Venegas, Dayhanne Alvear, Deyna Martínez, Diana Silva, Diego Abarca, Elías Fuentes, Elizabeth Inzunza, Fabián Alfaro, Fernanda Aqueveque, Fernanda Cartes, Fernanda Delgado, Fernanda Sandoval, Fernanda Tamayo, Francisco Espinoza, Gladys Espinoza, Gonzalo Inzunza, Gonzalo Vidal, Grisel Roca, Hileinn Sánchez, Jared Defaur, Jonathan Sazo, José Manuel Fuentes, José Miguel Cañete, Juan Pablo Vásquez, Karin Reyes, Karina Piña, Katherien Orellana, Lisandro Vega, Loreto Lagos, Magdalena Ponce, Catalina Maldonado, María Alejandra González, María Ignacia Torres, Mariana Irribarra, Mariangela Sanguinetti, Mario Leiva, Marjorie Ibacache, Martín Yañez, Martina Palamara, Massimo Magnani, Maykol Padilla, Millaray Arancibia, Milovan Acevedo, Génesis Morales, Nallely Castillo, Nélida Carvajal, Omar González, Paola Alvarado, Pía Muñoz, Renata Erazo, Rocío Silva, Rodrigo Sepúlveda, Rodrigo Valdés, Ronny Molina, Saraí Costa, Sebastián Alvear, Sofía Acuña, Sofía Mendoza, Sofia Sáez, Sofía Tapia, Tamara Cerda, Tomás Zamorano, Valentina Araya, Valentina Cortez, Valentina Pereira, Valentina Pino, Victoria Yáñez, Viviana Jaramillo, Yavanna Rivera, Yerko Urbina, Zuleimy Uzcátegui, Rodrigo A. Gutiérrez, Ariel Orellana, Martín Montecino, Alejandro Maass, Mauricio González, Miguel L. Allende, Christian Hodar, Paula Irles

**Affiliations:** 1 Millennium Institute Center for Genome Regulation, 7800003 Santiago, Chile; 2 Facultad de Ciencias, Universidad de Chile, 7800003 Santiago, Chile; 3 Facultad de Química y Biología, Universidad de Santiago de Chile, 9170022 Santiago, Chile; 4 INTA, Universidad de Chile, 7830490 Santiago, Chile; 5 Advanced Center for Chronic Diseases (ACCDiS), Sergio Livingstone 1007, 8380494 Independencia, Santiago Chile; 6 Facultad de Ciencias de la Vida, Centro de Biotecnología Vegetal, Universidad Andres Bello, Santiago, Chile; 7 Facultad de Medicina y Facultad de Ciencias de la Vida, Universidad Andres Bello, Santiago, Chile; 8 Institute of Agri-food, Animal and Environmental Sciences, Universidad de O´Higgins, Rancagua, Chile; 9 ANID-Millennium Science Initiative Program—Millennium Nucleus for the Development of Super Adaptable Plants (MN-SAP), Santiago, Chile; 10 Facultad de Agronomía e Ingeniería Forestal, Pontificia Universidad Católica de Chile, Santiago, Chile; 11 Facultad de Ciencias Biológicas, Pontificia Universidad Católica de Chile, Santiago, Chile; 12 Facultad de Ciencias, Ingeniería y Tecnología, Universidad Mayor, Santiago, Chile; 13 Departamento de Ingeniería Matemática, Facultad de Ciencias Físicas y Matemáticas, Universidad de Chile, Santiago, Chile

**Keywords:** *Euborellia annulipes*, *Forficula auricularia*, Nanopore sequencing, Citizen Science

## Abstract

**Background:**

Despite representing the largest fraction of animal life, the number of insect species whose genome has been sequenced is barely in the hundreds. The order Dermaptera (the earwigs) suffers from a lack of genomic information despite its unique position as one of the basally derived insect groups and its importance in agroecosystems. As part of a national educational and outreach program in genomics, a plan was formulated to engage the participation of high school students in a genome sequencing project. Students from twelve schools across Chile were instructed to capture earwig specimens in their geographical area, to identify them and to provide material for genome sequencing to be carried out by themselves in their schools.

**Results:**

The school students collected specimens from two cosmopolitan earwig species: *Euborellia annulipes* (Fam. Anisolabididae) and *Forficula auricularia* (Fam. Forficulidae). Genomic DNA was extracted and, with the help of scientific teams that traveled to the schools, was sequenced using nanopore sequencers. The sequence data obtained for both species was assembled and annotated. We obtained genome sizes of 1.18 Gb (*F. auricularia*) and 0.94 Gb (*E. annulipes*) with the number of predicted protein coding genes being 31,800 and 40,000, respectively. Our analysis showed that we were able to capture a high percentage (≥ 93%) of conserved proteins indicating genomes that are useful for comparative and functional analysis. We were also able to characterize structural elements such as repetitive sequences and non-coding RNA genes. Finally, functional categories of genes that are overrepresented in each species suggest important differences in the process underlying the formation of germ cells, and modes of reproduction between them, features that are one of the distinguishing biological properties that characterize these two distant families of Dermaptera.

**Conclusions:**

This work represents an unprecedented instance where the scientific and lay community have come together to collaborate in a genome sequencing project. The versatility and accessibility of nanopore sequencers was key to the success of the initiative. We were able to obtain full genome sequences of two important and widely distributed species of insects which had not been analyzed at this level previously. The data made available by the project should illuminate future studies on the Dermaptera.

**Supplementary Information:**

The online version contains supplementary material available at 10.1186/s40659-023-00414-9.

## Background

### Dermaptera: an underrepresented group within insect genomes

Insects are the most diverse group of animals, with more than one million species already named, though these represent less than 20% of the total estimated number of insect species [1]. Insects play fundamental roles in ecosystems, and strongly influence agricultural food production and human and animal health. Therefore, increasing our knowledge on the genetic and genomic underpinnings of their biology is fundamental. According to Li et al. [2], 1219 insect genome sequencing projects have been registered at BioProjects (NCBI) but, to date, only 401 species have had their complete genome sequenced. During the last decade, two global initiatives have been at the forefront of insect genome sequencing. One of them is the i5K [3]—and its workspace housed at the National Agriculture Library (NAL) [4]—that aimed to reach 5000 insect and arthropod genomes by 2015, but currently there are less than a tenth of what was expected. The InsectBase [5] is currently active and offers 817 insect genomes representing 20 orders. As expected, insect species of medical and agricultural interest have been prioritized, being well represented by orders such as Diptera, Lepidoptera, Hymenoptera and Coleoptera. Additionally, sequencing insect genomes has other difficulties that are related to the complexity in the analysis and assembly, including small sample material, high heterozygosity [2] and the large and highly repetitive nature of a major part of insect genomes [6], specifically in hemimetabolous animals [7, 8].

Dermaptera is a small insect order situated at the base of the Polyneoptera, the neopteran group of winged insects [9, 10]. It comprises close to 2000 extant species [11–13] grouped into 203 genera and 11 families [12, 14, 15]. The earwigs are distributed worldwide, and the highest number of species is found in the Tropics. In contrast, in temperate regions, such as Chile, a limited number of species have been recorded (c.a 20 species) [12, 16]. Earwigs correspond to hemimetabolous insects with 4–6 instar nymphs and morphologically recognizable characters such as forceps-like cerci at the end of the abdomen—the pincers -, and an elongated flattened body. Most of the species are oviparous, laying eggs in clutches. However, among earwigs two small families are viviparous and live non-parasitically associated with animals including bats and hamster rats [12] in which nymph survival is likely increased. Females display pronounced maternal care protecting eggs from external threats, typically predators and mold. There is ample documentation of maternal care behavior exhibited by these species to egg clutches and also to the first instar nymphs [17–21]. Individuals are nocturnal free-living, with omnivorous habits, feeding on plants or arthropods prey.

The European earwig *Forficula auricularia*, and the ring-legged earwig *Euborellia annulipes* are both cosmopolitan synanthropic species. *F. auricularia* is a subsocial, invasive univoltine insect species. But depending on climate a second brood can be found. Currently, 4-cryptic species have been identified in the Palaearctic region, mainly in Europe [22]. Like other dermapterans, *F. auricularia* has highly specialized wings despite being flightless. In contrast, *E. annulipes,* as are other species of Anisolabididae, are wingless [23]. These two are the most studied species in relation to their dual roles in the agroecosystem [24–26]. There is contrasting literature reporting the role and effect of these species in agriculture, acting as insect pests in grain, vegetable, and several fruit crops but also as biological control agents feeding on aphids, mites, psyllids, and other small arthropods [25, 27, 28]. Some studies performed in Australia have shown that *F. auricularia* is the most prevalent species feeding on grain crops [28] and has been reported to induce damage in several fruit species [26]. But Nicholas et al., [29] showed that *F. auricularia* in combination with the hymenopteran species, *Aphelinus mali,* were able to efficiently reduce woolly aphid infestations. In Europe, the beneficial role of *F. auricularia* in apple [30] and citrus orchards [31] has also been described, controlling sucking insect pests such as psyllids and aphids. In addition, *E. annulipes* has been studied in Brazil controlling eggs of armyworms and weevils [32, 33].

In terms of ovarian structure, earwigs have a meroistic polytrophic ovary, which means that ovarian follicles are made up of an oocyte-nurse cell complex, enveloped in a somatic follicular epithelium [34]. Particularly in earwigs, there is a single nurse cell in each growing ovarian follicle, compared to other species with this ovary type, such as *D. melanogaster*, which develops 15-nurse cells in each egg chamber. Based on a number of traits of the ovarian morphology, i.e., the number and length of ovarioles, the length of lateral oviducts, number of follicle cell populations and mitotic division of cystoblast, Tworzydlo [35] proposed two categories of ovaries, called as the “Anisolabis type” and the “Forficula type”. Their main differences are in the number and length of ovarioles and the length of the lateral oviducts. The Anisolabis type is representative for families of dermapterans considered basal [36]. This ovary is characterized by 5 elongated ovarioles with several developing germ cell cysts that later, during vitellogenesis, turn into larger ovarian follicles [21, 35]. In contrast, the Forficula type, characteristic of Eudermaptera, display many short ovarioles along an elongated lateral oviduct. Each ovariole comprises a short vitellarium with two ovarian follicles, as a consequence of a single mitotic division of the cystoblast [35].

The current phylogeny of earwigs, based on morphological characters such as ovary structure and orientation and number of penises, among several others, as well as molecular data [14, 15, 37–39], recognizes two major clades: the *Protodermaptera* (comprising the basal families Karschiellidae, Diplatyidae, and Pygidicranidae) and the *Epidermaptera* (comprising 8 families: Apachyidae, Labiduridae, Anisolabididae, Spongiphoridae, Arixeniina, Hemimerina, Chelisochidae and Forficulidae). However, the definitive phylogenetic relationships of the Dermaptera are not fully resolved. Among recent efforts to provide useful data, one study carried out sequencing of mitogenomic characters of four species of earwigs [40], and another, by Wipfler and coworkers [38], has carried out extensive and integrated phylogenetic analysis (combining massive numbers of nuclear genes with several morphological features). However, to date, there is only a single dermapteran species, *Anisolabis maritima* (Anisolabididae), whose genome was sequenced. In this sense, information on genomes of additional species have become necessary, and this study contributes with whole genome sequencing of two cosmopolitan earwig species, adding members of two additional families of the Epidermaptera, *F. auricularia* (Forficulidae) and *E. annulipes* (Anisolabididae).

### A genome project originated in the classroom

Among the first-hand experiences used to teach scientific concepts to school children, those that involve actual experimentation have proven to be highly motivating and influential in their behavior [41]. Most of these experiences involve predefined protocols or experiments that are aimed at emulating thought processes analogous to those of authentic scientific research. Others are original research projects, hypothesis driven initiatives that often lead to results and that can be presented at science fairs. Thirdly, there are many instances in which school students or communities engage in citizen science projects. In this case, a research project, usually led by a scientist, involves participation in field work, may require a wide geographical distribution of data collection or long-term following of a phenomenon. The results obtained in citizen science initiatives can be published and participants are often acknowledged as authors or contributors [42].

Since the sequencing of the first human genome in 2001, the cost per base of obtaining DNA sequence has decreased by several orders of magnitude [43]. Additionally, the technology required for sequencing nucleic acids has become increasingly accessible, even for non-specialists. An example in point is the availability of Oxford Nanopore’s MinIon sequencers, based on nanopore technology and a miniaturized platform, a system that has allowed sequencing in laboratories with a modest budget, for genomic analysis in the field [44, 45] and even in classrooms, though mostly beginning at the undergraduate level [46, 47]. The technology has also incorporated simplified steps for sample preparation and DNA purification which do not require expensive equipment or tools. Finally, many bioinformatic platforms are becoming available that allow the inexperienced user to perform some of the basic functions required to manage large numbers of sequence files. Thus, sequencing of nucleic acids (genomes) outside of the lab is feasible and can be a powerful way to engage the citizenry and disseminate knowledge on the power of genomics for human health, environmental protection, exploration of biodiversity and population genetics.

In 2018, five publicly funded Chilean Scientific Centers of Excellence (see Acknowledgements) launched the 1000 Chilean Genomes Initiative (www.1000genomas.cl) aimed at obtaining the full genome sequence of 1000 Chilean nationals and 1000 species that inhabit this country. That same year, the project became part of the global effort to sequence all eukaryotes, the Earth Biogenome Project [48]. Since the 1000 Chilean Genomes Initiative involves cutting edge science and genomics is a field with very relevant outcomes for the economy and quality of life of our fellow citizens, it considers the inclusion of a strong element of dissemination and outreach. We sought to launch the project by engaging the secondary school community on a nationwide level in order to illustrate how the new genomic era will be both accessible and pervasive throughout society. The school sequencing program was launched in 2018 with a second version held in 2019; further instances were interrupted by the COVID-19 pandemic. In both cases, we held a nationwide competition to participate in an original genome sequencing project and selected applications from different areas of the country favoring underrepresented populations and regions. The sequencing experiment was carried out simultaneously in all selected schools and the results were shared between participants through online platforms. Importantly, the participating students were aware that their work would become part of an original research effort that aimed to be published in a scientific journal.

**Fig. 1:**
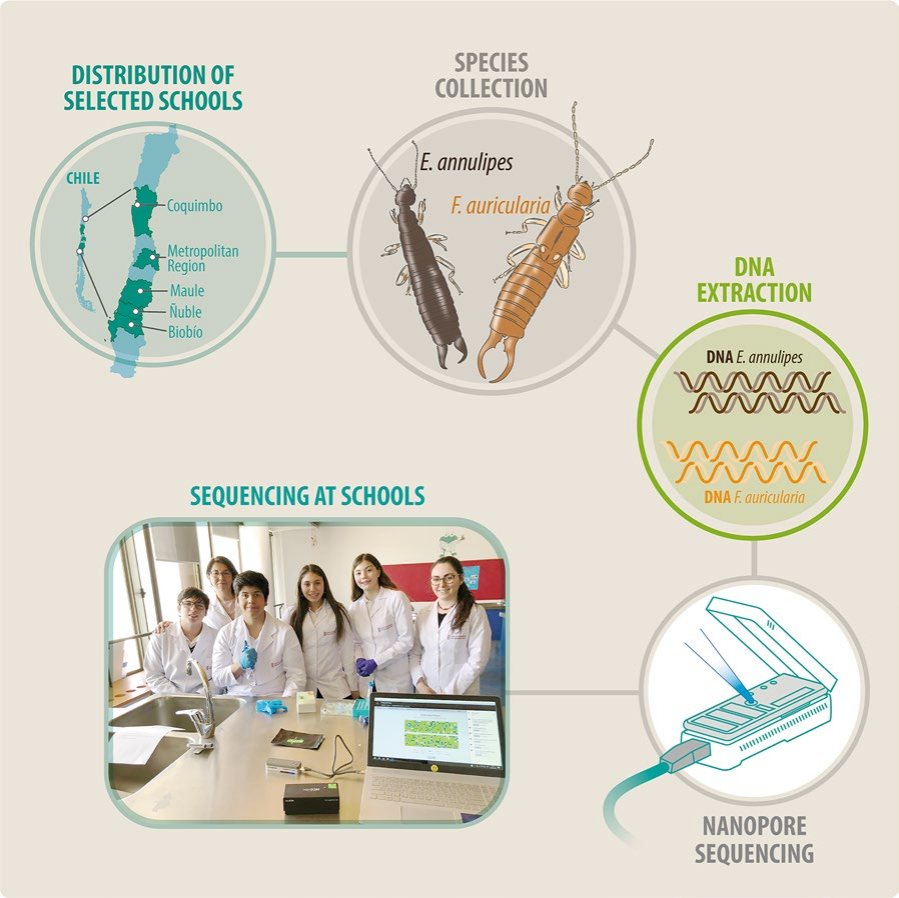
Schematic representation of the study. The distribution of the High Schools participating in the study along Chile is shown. Specimens of both earwig species, the European earwig *Forficula auricularia* and the ring-legged earwig *Euborellia annulipes*, were collected by students. Finally, DNA was extracted from the samples, and it was sequenced using nanopore technology at the high schools

In this article, we present the results of the school genome sequencing project held in 2019, in which the challenge was to collect and sequence DNA from common earwigs (insects of the order Dermaptera) found in the vicinity of the selected schools (Fig. 1). We obtained the complete genome sequence of two species, *Euborellia annulipes* and *Forficula auricularia* and we discuss the implications for genomics education and the characterization of this important group of insects.

## Results

### *Sequencing, base-calling and *de novo* genome assemblies*

For each species, genomic DNA from 15 individuals was sequenced using Oxford Nanopore MinIon sequencers (see Methods). General statistics of base-calling quality control are presented in Table 1. For both species, mean read length was around 3,000 base pairs (bp). The mean phred quality scores for these reads were 13.1 and 12.7 for *E. annulipes* and *F. auricularia* respectively. The total number of reads and the total number of bases sequenced for *E. annulipes* was 1.8 times bigger than those obtained for *F. auricularia*.

**Table 1:** General statistics of base-calling quality control

	*Forficula auricularia*	*Euborellia annulipes*
Mean read length	3055.8 bp	3006.7 bp
Mean read quality	12.7	13.1
Number of reads	6576750	11953319
Number of bases	20097322545	35940387157

We assembled de novo both genomes using the Flye software. The N50 (i.e., minimum contig length required to cover 50 percent of the assembled genome sequence) was larger in the *F. auricularia* assembly. Even though our coverage of the genomes was relatively low, for both species it was possible to retrieve more than 90% of complete insect core genes searched with BUSCO (93.3% *F. auricularia*, 97.1% *E. annulipes*). The total genome length was on the order of 1 gigabase (Gb), being slightly larger for the *F. auricularia* assembly (1.18 Gb vs 0.94 Gb) (Table 2).

**Table 2:** Genome assemblies’ statistics

	Total length	Number of fragments	N50	BUSCO completed^a^
*F. auricularia*	1186477850 bp	20519	150816 bp	93.3%
*E. annulipes*	944682793 bp	31851	47437 bp	97.1%

^**a**^Percentage of complete insect core genes found using BUSCO software (1)

### Structural and functional annotation

#### Interspersed repeats and low complexity DNA sequences

**Fig. 2:**
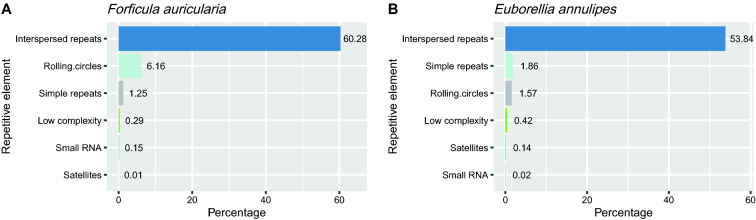
Repetitive sequence annotation. This graph shows the contribution of repetitive elements in percentage relative to the total number of Interspersed repeats and low complexity DNA sequences identified in the genomes of *F. auricularia* (**A**) and *E. annulipes* (**B**)

To initially characterize the earwig genomes, an ab initio repeat search was conducted with RepeatModeler [49] and the sequences were further classified with RepeatMasker [50]. For both species, the highest proportion is represented by interspersed repeats of the transposon and retrotransposon type comprising 60.28% and 53.84% of the genomes of *F. auricularia* and *E. annulipes*, respectively. Transposable elements using a "rolling circle" type of replication are in higher proportion (6.16%) in the genome of *F. auricularia* compared to that of *E. annulipes* (1.57%). Repetitive elements such as simple repeats, low complexity regions, small RNAs and satellite repeats comprise a small proportion of the repetitive sequences in both genomes and show small differences in terms of representation in the genome of both species (Fig. 2).

#### Non-coding RNAs

**Fig. 3:**
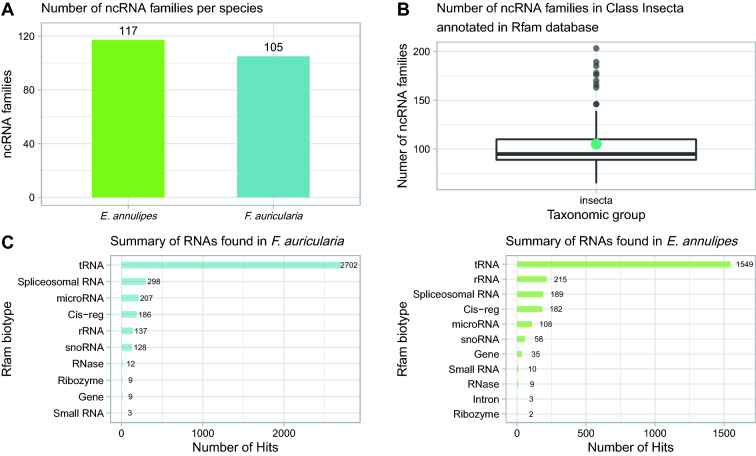
Non-coding RNA annotation. **A** This graph shows the number of non-coding RNA families identified in the genomes of *F. auricularia* and *E. annulipes*. **B** Distribution of the number of non-coding RNA families from 78 insect species, including *F. auricularia* and *E. annulipes. G*reen dot represents the mean number of ncRNA families. **C** This graph shows the number of the main ncRNA biotypes found in the genome of both earwig species

The Rfam database [51] classifies the different biotypes of non-coding RNAs (ncRNAs) into families according to multiple sequence alignments and consensus on their secondary structure. The number of ncRNA families annotated for *F. auricularia* is 105 versus 117 for *E. annulipes* (Fig. 3). The number of ncRNA families for both *F. auricularia* and *E. annulipes* falls within the interquartile range of the data present in the Rfam database, which represents 78 annotated insect species (Fig. 3B). In relation to the biotypes of ncRNAs, for both species, transfer RNAs (tRNAs) are the most abundant ncRNA biotype, which is consistent with being the most abundant gene family in the genomes (Fig. 3C).

**Fig. 4:**
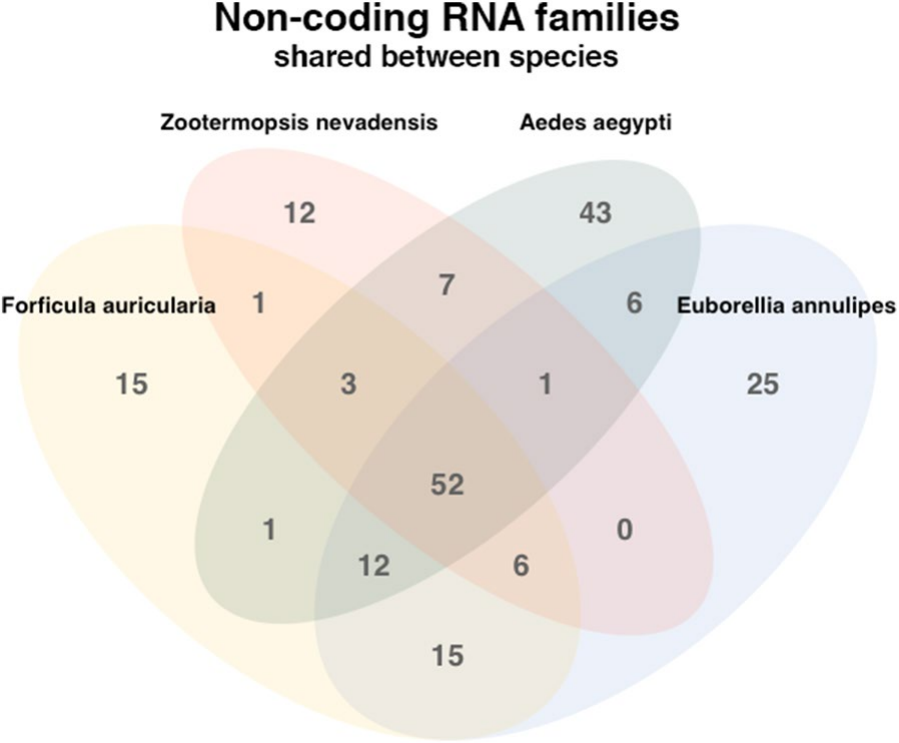
Venn diagram of Non-coding RNA families shared between species. The total number of unique and common ncRNA families between *F. auricularia, E. annulipes, Z. nevadensis* and *A. aegypti* is displayed

The number of ncRNA families shared between the studied species and the two more closely related insect species whose annotations were available in Rfam database, the dampwood termite *Zootermopsis nevadensis* and the yellow fever mosquito, *Aedes aegypti*, is shown in Fig. 4. From the total of ncRNA families (189), the number of ncRNA families shared among all species is 52, this number increases to 85 if only the families shared between *E. annulipes* and *F. auricularia* were observed, 15 of these families are shared exclusively by these two earwig species.

**Fig. 5:**
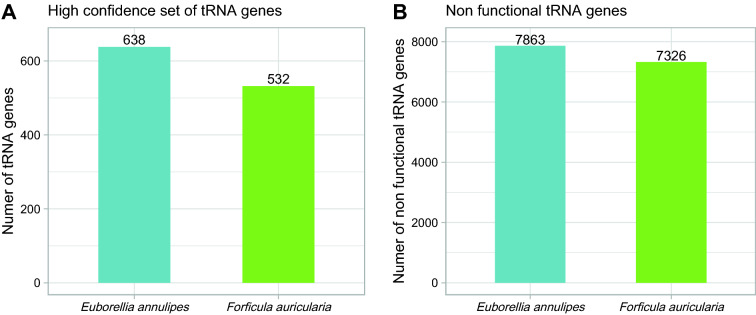
Transfer RNAs annotation. **A** Number of tRNA genes annotated with high confidence for both species. **B** Number of predicted nonfunctional tRNA genes annotated for both species

The annotation of transfer RNAs was carried out using the tRNAscan-SE software [52] given its higher accuracy for the annotation of these types of elements. A total of 8501 tRNA genes were estimated for *E. annulipes* and 7,858 for *F. auricularia*. Considering that there is a high number of tRNA pseudogenes in eukaryotic genomes, a postfiltering tool included in tRNAscan package was used to determine that set of genes that, with high confidence, are involved in translation. In Fig. 5A, the number of tRNA genes annotated with “high confidence” is shown, where *E. annulipes* presents 106 more genes than *F. auricularia* (638 versus 532 tRNA genes). The annotated non-functional tRNAS (Fig. 5B) account for 92.5% and 93% of all tRNA gene annotations of *E. annulipes* and *F. auricularia*, respectively.

#### Structural annotation of protein coding genes

The main results of the structural gene annotation performed with the BRAKER2 pipeline [53] are detailed in Table 3 (for complete statistics refer to Additional file 4). *E. annulipes* had 8,249 more genes than *F. auricularia*, with a total of 40,028 predicted protein coding genes, which represent 26.18% of the total genome in base pairs. The genome of *F. auricularia* showed 31,779 protein coding genes, which represent 26.53% of its genome in base pairs.

**Table 3:** Structural annotation of protein coding genes for both earwig species

	*Forficula auricularia*	*Euborellia annulipe*s
Number of genes	31779	40028
Number of CDSs	33587	42675
Number of exons	147010	198427
Number of introns	117244	162278
Total gene length	310662.7 kb	250644 kb
Total CDSs length	30234.7 kb	44792.7 kb
Mean gene length	9.8 kb	63 kb
Mean CDSs length	0.9 kb	1.049 kb
Mean exon length	293 bp	297 bp
Mean intron length	2821 bp	1578 bp
Mean 5′ UTR length	3454 bp	2351 bp
Mean 3′ UTR length	3274 bp	1921 bp

**Fig. 6:**
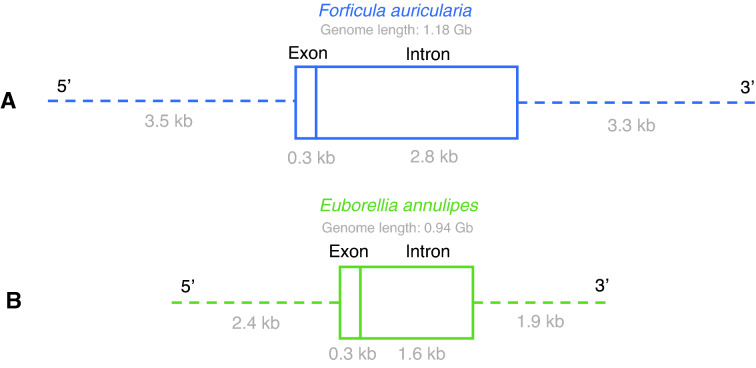
Schematic representation of mean gene length. **A** *Forficula auricularia* **B** *Euborellia annulipes*

Although *E. annulipes* has a greater number of genes, the total length of these genes measured in base pairs is smaller compared to *F. auricularia*. This difference can be explained by a greater total length of introns and a greater average length of introns in the case of *F. auricularia* (Fig. 6), as well as by the average length of the 5' and 3' UTR regions in *F. auricularia*, which are 1,103 and 1,353 bp longer, respectively, than those regions in the genome of *E. annulipes*. The number of single exon genes was higher in the case of *E. annulipes*, outnumbering *F. auricularia* by 1,151 genes. On the other hand, the average number of introns and exons per mRNA was slightly higher in *E. annulipes* compared to *F. auricularia*.

#### Functional annotation of protein coding genes

Using the Swissprot database [54], 58.4% and 59.9% of the total proteins of *F. auricularia* and *E. annulipes*, respectively, were annotated. When using the insect protein database extracted from NCBI, a higher percentage of proteins was annotated for both species, with 67.5% of the proteins annotated for *F. auricularia* and 65.4% for *E. annulipes*. The annotation of orthologs performed with the EggNOG database [55], identified 30,360 orthologs for *E. annulipes*, which corresponds to 71% of the total structurally annotated sequences, and 22,800 orthologs for *F. auricularia*, which corresponds to 68% of the structurally annotated sequences. Of all annotated orthologs, 40% [17] of *E. annulipes* and 44% (14,785) of *F. auricularia* had Gene Ontology (GO) term annotations. Both species share 8,027 of them and considering those represented more than once in each genome, *F. auricularia* shares 57% of its orthologs with *E. annulipes* and *E. annulipes* shares 50% with *F. auricularia*.

### Functional comparative analysis

#### Orthogroup analysis

Table 4 details the overall results from Orthofinder, also considering the proteomes of 8 species belonging to the winged insect group Pterygota (see details of species in methods). In total, more than 300,000 genes from these species were analyzed, of which 239,995 are present in orthogroups, representing 78.2% of all input genes. These genes were grouped into 29,794 orthogroups of which 4,449 were present in all species, and exclusive orthogroups (species-specific) were 9,584 in total.

**Table 4:** General statistics of orthogroup analysis with Orthofinder

General statistics	
Number of species	10
Number of genes	302979
Number of genes in orthogroups	239995
Percentaje of genes in orthogrups	79.2%
Number of species-specific orthogroups	9584
Ortogrups with all species present	4449
Mean orthogroup size	8.1

Table 5 summarizes the main results focused on the two species under study. For both species, more than 80% of their genes were assigned to orthogroups, this value being slightly higher for *Euborellia annulipes*. These genes were grouped into 14,366 orthogroups in the case of *Forficula auricularia*, and 17,063 other groups in the case of *Euborellia annulipes*. Of all the orthogroups, 866 were found exclusively in *Forficula auricularia*, comprising 3,372 genes corresponding to 10% of its structural annotation. *Euborellia annulipes*, on the other hand, presented 1,839 exclusive orthogroups comprising 8,425 genes, which represented 19.7% of its structural annotation. Both species are present in 12,226 orthogroups in conjunction with other species, and of these 1,092 orthogroups are unique to *F. auricularia* and *E. annulipes* together.

**Table 5:** Species-specific statistics from orthogroup analysis with Orthofinder

	*F. auricularia*	*E. annulipes*
Number of genes	33587	42675
Number of genes in orthogroups	28123	38298
Percentage of genes in orthogroups	83.7%	89.7%
Percentage of unassigned genes	16.3%	10.3%
Number of orthogroups containing species	14366	17063
Number of species-specific orthogroups	866	1839
Number of genes in species-specific orthogroups	3372	8425
Percentage of genes in species-specific orthogroups	10%	19.7%
Number of shared orthogroups with other species	12226	12226
Number of shared othogroups only between species	1092	1092

#### Enrichment of GO terms

As for the Gene Ontology term enrichment analysis, 5,034 GO terms corresponding to genes present in orthogroups exclusive to *F. auricularia* were analyzed, of which 356 (Additional file 1) were found to be enriched with the parameters as described in Materials and Methods. In the case of *E. annulipes*, 6,401 GO terms were analyzed, of which 350 were found to be enriched (Additional file 2). A subset of the most relevant GO terms enriched in each of the two species can be seen in Tables 6 and 7, which include the categories biological processes, molecular functions, and cellular compartments.

**Table 6:** Top 15 GO terms for biological processes, molecular functions and cellular compartments enriched in species specific orthogroups of *Forficula auricularia*

*Forficula auricularia*			
GO ID	Adj. p-value	Parent term	Term
GO:0070725	4.11E-11	Intracellular anatomical structure	Yb body
GO:1990923	4.22E-11	Protein-containing complex	PET complex
GO:2000241	6.53E-11	Reproductive process	Regulation of reproductive process
GO:0006313	6.53E-11	DNA recombination	Transposition DNA-mediated
GO:0046012	6.53E-11	Regulation of translation	Positive regulation of oskar mrna translation
GO:0042078	2.19E-10	Germ cell development	Germ-line stem cell division
GO:0090101	2.93E-10	Negative regulation of signal transduction	Negative regulation of transmembrane receptor protein serine/threonine kinase SP
GO:0051321	3.30E-10	Reproductive process	Meiotic cell cycle
GO:0017148	3.30E-10	Regulation of translation	Negative regulation of translation
GO:0036369	3.70E-10	Proteasomal protein catabolic process	Transcription factor catabolic process
GO:0000153	5.15E-10	Catalytic complex	Cytoplasmic ubiquitin ligase complex
GO:0032196	5.36E-10	Cellular process	Transposition
GO:1903046	5.37E-10	Reproductive process	Meiotic cell cycle process
GO:0050779	5.65E-10	Regulation of RNA stability	RNA destabilization
GO:0034249	9.14E-10	Nitrogen compound metabolic process	Negative regulation of cellular amide metabolic process

**Table 7:** Top 15 GO terms for biological processes, molecular functions and cellular compartments enriched in species specific orthogroups of *Euborellia annulipes*

*Euborellia annulipes*			
GO ID	Adj. p-value	Parent term	Term
GO:0044771	3.59E-20	Reproductive process	Meiotic cell cycle phase transition
GO:1901993	1.06E-19	Reproductive process	Regulation of meiotic cell cycle phase transition
GO:1905134	1.06E-19	Reproductive process	Positive regulation of meiotic chromosome separation
GO:1905820	1.61E-19	Cell cycle process	Positive regulation of chromosome separation
GO:0051305	5.76E-19	Cell cycle process	Chromosome movement towards spindle pole
GO:1905132	1.01E-18	Reproductive process	Regulation of meiotic chromosome separation
GO:0045836	1.18E-18	Reproductive process	Positive regulation of meiotic nuclear division
GO:0016344	2.72E-18	Reproductive process	Meiotic chromosome movement towards spindle pole
GO:0045752	3.29E-18	Regulation of signal transduction	Positive regulation of Toll signaling pathway
GO:0050000	3.29E-18	Organelle localization	Chromosome localization
GO:0002804	3.57E-17	Defense response to fungus	Positive regulation of antifungal peptide production
GO:0051446	3.57E-17	Reproductive process	Positive regulation of meiotic cell cycle
GO:1901995	3.57E-17	Reproductive process	Positive regulation of meiotic cell cycle phase transition
GO:0003746	7.61E-17	Regulation of translation	Translation elongation factor activity
GO:1900150	3.57E-17	Defense response to fungus	Regulation of defense response to fungus

Given the number of enriched GO terms and the interest in focusing on those that reveal enriched biological processes, we use Revigo [56] to select the terms that are most representative of the group analyzed, forming clusters of GO terms considering their p-value values and their GO category.

**Fig. 7:**
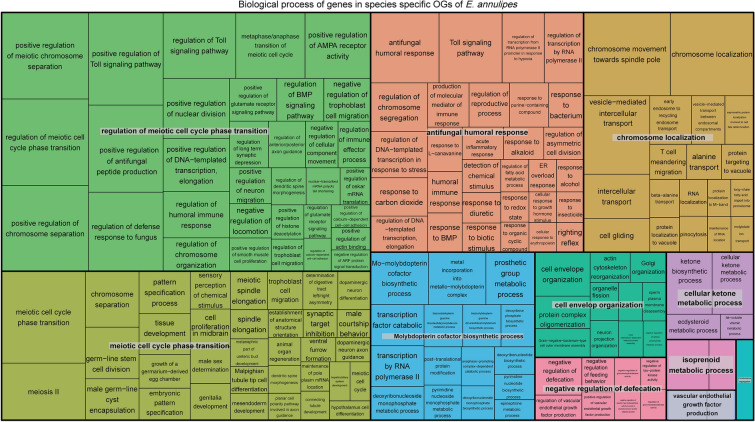
Treemap of biological processes enriched in species-specific orthogroups of *E. annulipes*. Each rectangle represents a group of closely related GO terms with a “cluster representative” giving the name of the cluster. The representatives are then grouped together into “superclusters” of loosely related terms (same color). The size of each rectangle represents the adjusted p-value of the cluster representative

In the case of *E. annulipes,* the biological processes enriched species-specifically in orthogroups coalesced into the categories of “Regulation of meiotic cell cycle phase transition”, “Meiotic cell cycle phase transition”, “Humoral antifungal response”, “Chromosomal localization”, among others (Fig. 7).

**Fig. 8:**
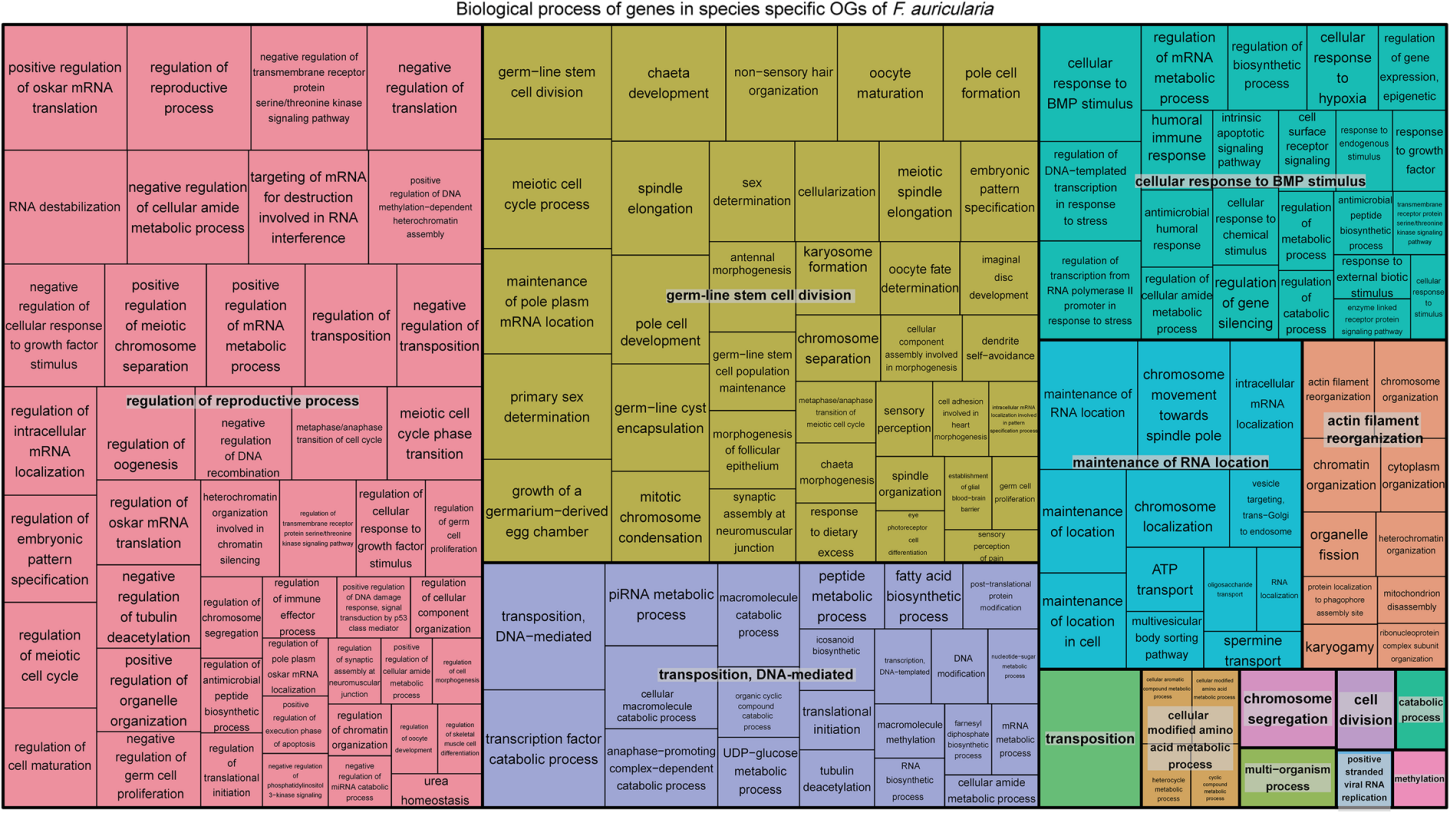
Treemap of biological processes enriched in species-specific orthogroups of *F. auricularia.* Each rectangle represents a group of closely related GO terms with a “cluster representative” giving the name of the cluster. The representatives are then grouped together into “superclusters” of loosely related terms (same color). The size of each rectangle represents the adjusted p-value of the cluster representative

As for *F. auricularia*, the enriched biological processes were grouped in the categories of “Regulation of the reproductive process”, “Germline stem cell divisions”, “Transposition, DNA-mediated”, “Cellular response to BMP stimuli”, “Maintenance of RNA localization”, among others (Fig. 8).

## Discussion

Just as classrooms evolve with new technologies for learning, science education must evolve to familiarize new generations early with the scientific principles that will drive society in the coming decades, including access to genetic information and the emerging technologies in DNA manipulation. Historically, genome sequencing has been a process that requires sophisticated instruments and must be carried out in a laboratory. However, thanks to the development of new technologies, it is now possible to perform in situ DNA sequencing in places as remote as the equatorial jungle [57], the polar territories [58, 59], on the International Space Station ISS [45], as well as in more accessible places such as a classroom [46, 47]. In this manuscript, we described the analysis of two earwig genomes obtained through an interaction of a research team with high-school students from five regions of central and southern Chile. School students participated in the collection of the earwigs, identified the sampled animals, and carried out the sequencing work in their schools in a synchronously coordinated experience. Thus, they became first-hand participants in an actual scientific endeavor, one that was highly collaborative and multidisciplinary. Furthermore, the school students have also been able to see the project through its completion, manifested in a publication of scientific and social interest. Our evaluation of the experience among students and teachers indicated that it has had a significant impact on motivation, their understanding of the science involved, their standing among their peers and on their future career choices.

Once the earwig genomic sequences were obtained and collected in a single sequence pool for each species, an *in-silico* comparison was performed that began with a genome assembly. The quality of the generated assemblies was evaluated using complementary metrics such as the BUSCO tool [60]. This allowed us to assess the integrity of the genomes in terms of the expected genetic content based on the search for single-copy orthologs found in at least 90% of the species included in the group, in this case, insects. For both species, more than 93% copies of these complete single-copy orthologs were found (93.3% *F. auricularia* and 97.1% *E. annulipes*). For assemblies of non-model species, Seppey and colleagues [60] report completeness rates between 50–95%, and for model species over 95%. In this sense, the assembly obtained for *F. auricularia* was positioned at the upper limit of what could be expected and that of *E. annulipes* exceeded these expectations, indicating an integrity of the assemblies in a biological-evolutionary sense that provided a high level of confidence to continue with a comparative analysis of the genomic content of both species.

There is limited information about the genome sizes of the various groups of insects, however it can be stated that genome size depends on the evolutionary position within insect phylogeny, which somewhat reflects their life history and post-embryonic development [6]. When we began the earwig genome project in 2019, there was no available genome from any Dermaptera species, but recently the genome of *Anisolabis maritima* (Anisolabididae) was uploaded/released by the InsectBase platform. Compared to our data, the genome of *A. maritima* (649.7 Mb) is smaller than the genome sizes of *F. auriculari*a (1.18 Gb) and *E. annulipes* (0.94 Gb). These differences could be explained, among other factors, by the number of repetitive sequences present in the genomes of these species. This is the case between the two earwig sequences as *F. auricularia* exhibits 68.15% repetitive sequences versus 57.84% of *E. annulipes*; the difference of 206 Mb in favor of *F. auricularia* is represented mainly by transposable elements (TEs).

The analysis of TEs in insect genomes has shown that this diverse group of animals displays a great variability in the fraction of the genome that these elements occupy: from 11% in the fly *Drosophila simulans* to 93% in the green drake mayfly *Ephemera danica*; with an average of 56% [61]. Among hemimetabolous insects, the German cockroach *Blattella germanica* and the drywood termite *Cryptotermes secundus*, show genomes containing 55% of repetitive content, being the LINEs the most abundant transposable elements [7]. The TE content of a genome is based on a balance between the TE acquisition rate, their replication dynamics within the genome and their deletion rate [61]. The acquisition of these elements in the genome occurs by vertical inheritance, as they are inherited from ancestors, and by horizontal inheritance from other organisms. These species diverged approximately 160–140 million years ago [62], so the difference observed in the number of TEs could be attributed to the transposition process itself, by their deletion rates, and/or by the horizontal acquisition of these elements. Peccoud et al. [63], position horizontal inheritance of TEs as a force of great importance for the evolution of insect genomes, stating that horizontally transferred TEs generated up to 24% (2.08% on average) of all nucleotides in the genomes of these animals [63].

Regarding the annotation of non-coding and transfer RNAs, it was observed that both species had a similar number of ncRNA families. When this number was compared to the number of families of ncRNAs of 78 species of insects, both *F. auricularia* and *E. annulipes* were found in the interquartile range of the data obtained, with the lowest value being 65 families in the mosquito *Anopheles quadriannulatus* and the highest value 200 families in the fruit fly *Drosophila melanogaster*. Extending this comparison to the type of families of non-coding RNAs that the dermapterans presented, it was possible to see that when analyzed together with two species of Pterygota insects—*Zootermopsis nevadensis* and *Aedes aegypi*—all four species shared 52 families and this number increased to 85 if we restricted the comparison to those families only shared by *E. annulipes* and *F. auricularia*. These results were consistent with the fact that these species belong to the same order and therefore it is expected that they have greater genomic coincidences with each other than with more distant species.

The tRNAScan software predicted 8501 and 7858 tRNA genes for *E. annulipes* and *F. auricularia*, respectively. As part of the functional classification process, this program evaluates tRNA gene predictions to identify possible pseudogenes based on characteristics commonly observed in non-functional tRNAs [52]. This is because in many eukaryotic genomes, SINE retrotransposons derived from tRNA genes are numerous. Of all predictions, only 638 (*E. annulipes*) and 532 (*F. auricularia*) correspond to "high confidence" genes, the rest likely being non-functional tRNA genes. Comparing these numbers inside the 18 insect genomes annotated with "high confidence" in the Genomic tRNA database [64] the number of tRNA genes in the earwig's genomes was similar to the lepidopteran species *Spodoptera frugiperda*, which represents the species with the highest number of confident genes; and quite elevated compared to the only 196 genes found in the termite, *Zootermopsis nevadensis*.

We found that, for both species analyzed, 26% of their genome corresponds to protein coding genes. *E. annulipe*s showed a greater number of genes, surpassing *F. auricularia* by 8,249 genes (40,028 vs 31,779) and by 9,088 coding sequences. However, the average length of genes measured in base pairs of *F. auricularia* far exceeds that obtained for *E. annulipes*, which is explained in part by a greater average length of introns and of the 3' and 5'UTR regions in the first species, as well as by a greater number of single exon genes in *E. annulipes*. Carrying out a comparison at the protein level, it was observed that *F. auricularia* shares 35.6% of its proteins with *E. annulipes*, and *E. annulipes* shares 31.6% of its proteins with *F. auricularia*. Importantly, while both species belong to the same order, they are not closely related to each other evolutionarily. This is supported by the phylogenetic history described for the order, in which *E. annulipes* belongs to the Anisolabididae family linked to the clade Epidermaptera. In turn, *F. auricularia* belongs to the Forficulidae family that subsequently derived to the well-supported Eudermaptera clade [38]. One of the characteristic morphological traits is the ovary morphology and number and the orientation of penises among earwigs. Families of basal Protodermaptera lineage have two penises, both posteriorly oriented [65, 66]. Whereas males of Epidermaptera have typically two pennises but one of them oriented to posteriorly and the other to anteriorly, and derived Eudermaptera the presence of a single penis is considered as an apomorphy character [38].

We carried out functional annotation with two different software. Of the structurally annotated proteins generated by EggNOG-Mapper, 71% of *E. annulipes* and 68% of *F. auricularia* proteins were annotated as orthologs. These percentages are lower when compared to genes assigned to orthogroups we used the Orthofinder software [67], which reach 89% for *E. annulipes* and 83% for *F. auricularia.* Although both softwares use phylogenetic trees to predict orthologs, the difference is that Orthofinder uses a comparison between assigned species, in this case 10 species of Polyneoptera lineage, in addition to including paralogous and orthologous genes within the orthogroups. Instead, EggNOG-Mapper makes use of a database that includes not only insects, but only classifies orthologous genes [68].

The analysis carried out with Ontologizer allowed us to find 356 enriched GO terms for *F. auricularia*, and 350 for *E. annulipes* belonging to the three GO categories. We focused on those terms from the enrichment analysis that represent biological processes and it was observed that, in the case of *E. annulipes,* there was a clear predominance of terms related to the regulation of meiosis such as "Meiotic cell cycle transition", "Meiosis II", “Positive regulation of meiotic chromosome separation” among others. Although *F. auricularia* also presented enriched terms related to meiosis, these were less common and, as they belong to specific orthogroups of each species, come from different proteins. In fact, in *F. auricularia,* a large number of enriched biological processes related to the categories of regulation of the reproductive process were found, including “Regulation of oocyte development”, “Regulation of reproductive process”, “Regulation of germ cell proliferation” and, secondly, processes related to transposition, where “Transposition, DNA mediated”, “piRNA metabolic process”, among others, stand out.

These enriched biological processes found in our analysis become relevant when we examine the structural and cellular morphology of the ovary in the earwig species. Ovariole number varies vastly across insects but it is one of many other factors that determine fecundity [69]. Ovaries of basal dermapterans, such as *E. annulipes,* correspond to the “Anisolabis type” [35] having a few elongated ovarioles with up to 30 potential ovarian follicles. Usually, they develop up to 8 follicles per ovariole that finally turn into clutches of 30 eggs on average [18, 21]. In *F. auricularia* ovaries, representing the “Forficula type” [35], there are several short ovarioles with two ovarian follicles each and the clutch size varies from 16 to 40 eggs [19, 70]. These morphological characters may be related to the type of voltinism found in each of the studied species. *E. annulipes* is a polyvoltine species with several generations a year while *F. auricularia* is generally considered to be univoltine (single-brood populations). However, *F. auricularia* currently is a complex of sibling species in which some populations develop a second nest in the season (double-brood populations) [70–73].

The morphological differences in the structure of dermapteran ovaries are also established at the cellular level. In insects, the final number of germline cells contained in the cysts is highly variable and specific both at the species and group levels, depending on the number of consecutive divisions that the stem cell undergoes [34, 74]. Once more, in earwigs the development of ovarian follicles differs between basal and derived species within the Dermaptera order. In *E. annulipes,* cystoblasts divide three times, generating eight-cell cysts that then split into 4-cell and 2-cell cysts. The ontogenic events that lead to oocyte-nurse cell complex in the “Anisolabis type” are unique among insects with meroistic polytrophic ovaries because of the occurrence of a secondary division of the germline cysts [34–36]. In more derived taxa, including *F. auricularia*, the stem cells divide only once, skipping the intermediate 8-cell stage [35, 75].

The set of biological processes that we have found to be enriched in the genomes of the two sequenced species are indicative of the specific biological adaptations that have occurred in these lineages. In *F. auricularia* these processes were related to broad reproductive processes (regulation of reproduction and germ-line stem cell division) followed by transposition and DNA-mediated processes. In *E. annulipes*, they were clearly associated with regulation of the meiotic cell cycle and with the humoral immune defense response. Thus, it would be worthwhile to perform comparative studies between these two earwig species considering the differences found in terms of ovarian morphology and the ontogeny of oogenesis, as well as the molecular mechanisms under the humoral immune response in response to maternal care.

## Conclusions

This work is a pioneering experience that, using state-of-the-art mobile DNA sequencing technologies, brings school students closer to the generation of cutting-edge genomic knowledge. In addition, this type of initiative brings the scientific community closer to the schools and their communities, promoting the country's scientific development. Each schoolchild was the protagonist of the acquisition of an unpublished genomic resource generated from a joint collection of specimens they observe daily in their gardens, and they will now be able to see it differently thanks to the genomic knowledge they have generated. Through a citizen science project, the genomes of two species of earwigs have been sequenced, assembled, and annotated. Obtained genomes are of high-level confidence with a draft-level genome continuity that comprises more than 93% of single-copy orthologs genes from the insect group. Both species represent relatively large genomes where *F. auricularia* was larger than *E. annulipes*, but the last one with a major percentage of repetitive elements, represented mainly by transposable elements (TEs). In addition, 26% of both genomes are coding genes, with high similitude in non-coding and transference RNA families. At the biological level, *F. auricularia* presented an enriched set of protein orthogroups related to geminal cells and reproductive processes compared with *E. annulipes*, unique biological features that may have played a role in their evolutionary history.

This research represents a first insight into the genomic understanding of these species, which, through a genetic approach, has shed light on the similarities and differences present in the genomes and their enriched biological processes. Furthermore, this work allows further research on proteins related to reproduction and germ cell production, which are differentially represented in the genome of *F. auricularia* and investigates the evolutionary significance of the transposable elements present in these species.

## Design and methods

### Secondary school sequencing project

#### a. Selection of participants

Planning the school competition for participation in the sequencing activity took about 6 months. First, an organizing team of scientists was assembled (the main authors of this study). The application instructions and requirements were generated, a flier announcing the activity was produced (Additional file 3) and a web page was created (www.1000genomas.cl), which provided information, materials for application and contact details. Social media platforms were used: Twitter, Facebook and Instagram accounts were announced and promoted in networks related to science, science outreach and education. Dissemination of the competition guidelines was done using the country-wide network of EXPLORA, the branch of CONICYT (the Chilean Science Agency) tasked with outreach. Among the requirements for applying, we asked each candidate group to be composed of a maximum of 10 high school students and their science teacher. With the application, we requested an essay detailing why they were interested in participating and to provide evidence of previous scientific activities in their school. The applicants also had to provide written permission from the school principal and written consent from parents/guardians of all minors. To select the groups that would carry out the experiment, the scientific centers of excellence backing the initiative nominated a panel of five judges (one scientist from each of the five centers) who reviewed the applications and chose 12 of them to carry out the experiment. Among the criteria used were quality of the essay provided, and evidence of previous involvement in scientific activities. In addition, preference was given to public institutions and to those from regions outside the capital metropolitan area. There was also an effort to ensure gender balance among the students. After announcement of the competition results, all of the applicants agreed to participate and they were informed of the schedule for preparation of the experiment.

#### b. Preparation of the experiment

The participants were instructed to search for and collect individuals of the two most common species of earwigs (Dermaptera) found throughout Chile. A field guide describing the two species of interest: the ring-legged earwig, *Euborellia annulipes* and the European earwig, *Forficula auricularia* was created and given to participants to identify and properly collect the specimens. These species have been introduced into the country and are thereby not threatened or protected; there is no restriction on their capture and use according to local authorities (Servicio Agrícola y Ganadero, Ministerio de Agricultura, Chile). However, Chilean law prohibits the use of live animals for experimentation within elementary or secondary school property (Ley 20.380, 2009). Therefore, we could not carry out the entire experiment on site. Three weeks before the experiment was to be carried out, we mailed a packet to each participating school team containing 50 ml plastic Falcon tubes, latex gloves and a set of instructions. Students were instructed to collect between 5 and 10 animals in an area near the school, to georeference the collection sites and to photograph both the location and the animals with as much detail as possible. The specimens and data were sent to the Bioinformatics and Gene Expression Laboratory of INTA—University of Chile, where species identification was confirmed, and DNA extraction was performed. DNA preparation and quality control tests were performed to make sure DNA was of sufficient purity for sequencing; our research team carried out a sequencing run with each sample prior to performing the experiment in the schools to guarantee its success.

For sequencing, we used Oxford Nanopore Technologies’ (Oxford, UK) MinIon sequencing platform. We used one flow cell per participating school. MinIon sequencers and Rapid Sequencing Kits were provided as a gift by Oxford Nanopore Technologies. We also acquired 10 laptop computers for coupling to the MinIon sequencers and to collect the data. These were HP computers with 12 Gb of RAM and 512 Gb of SSD disk as required by Oxford Nanopore’s proprietary software. We attached to the computers a webcam so that each participating group could communicate with the scientists at the University on the day of the event and for live streaming of the experiment on the web. To have all the materials needed for successfully carrying out the experiments on site, we purchased the required molecular biology reagents, plasticware, micro pipettes, gloves, solutions, magnetic stands, tube racks and lab coats for all participants (10 complete sets of materials stored in suitcases and provided to each team of instructors).

#### c. Training and selection of instructors

Each participating school was to receive the visit of two instructors who would guide the experiment and who had sufficient knowledge of the concepts and methods to answer all inquiries. Since the experiment was to be carried out simultaneously in all locations distributed along the country, we needed a minimum of 20 instructors. Again, these were recruited from the five centers of excellence and were, for the most part, graduate students or postdocs with training in molecular biology and bioinformatics. As not all the instructors were versed in the use of the Nanopore sequencers, we held three training sessions where we covered library preparation, priming and loading the flow cells, running the MinIon sequencer, evaluating performance and observing the rate of sequencing in real time. Since the optimal time for the sequencing run is 24 h, we planned for a two-day experiment in which reactions were carried out and sequencing was begun on day 1, while the result would be obtained on day 2. As there was ample time on both days without any activities, we prepared a presentation and several exercises aimed at teaching molecular biology and genomics concepts; all instructors were trained for these activities as well. In addition, we asked all participating school teams to prepare a presentation of their own in which they described the experience of collecting biological samples in the field, to research the characteristics of the organisms to be sequenced and to hypothesize on what could be learned from their genomes.

Finally, the organizing team took care of the logistics of sending the 20 instructors to their respective destinations by providing airline or bus reservations, obtaining lodging and local transportation at each site. On the day before travel, the instructors collected the materials which included reagents and the sequencing flow cells that were to be kept cold in ice packs and stored refrigerated on site.

#### d. Sequencing in the schools

To generate interest among the general public for this activity, we carried out a promotional campaign to inform the press and communicators at different organizations involved with science and education. Since the experiment was to be performed simultaneously in all schools, we coordinated availability of teachers and students. All groups were instructed to end the sequencing run at a specific time on the second day in order for each one to inform the result obtained through a live video streaming transmission. Some schools did not have adequate internet availability; in those cases, we provided instructors with data dongles for connection to the cellular network. The experiment with the school students was carried out on September 26 and 27 of 2019.

#### e. Follow-up and evaluation of impact

All instructors were asked to carry out interviews of teachers and students during the two days of the experiment. A questionnaire was prepared in order to have a systematic way in which to organize the responses. Interviews were recorded on video and all material was recovered in a centralized cloud account. Two weeks after the event, a survey (generated in Google Forms) was sent to all participating teachers and students to obtain further information on the impact of the experience. We will report on the results of this aspect of the experiment elsewhere.

### Material preparation, sequencing, and analysis

#### a. DNA extraction and sequencing

DNA extraction was performed using anterior (head and antennae) and posterior (forceps) appendages using 3 specimens per sample, sequencing 5 samples per species. The E.Z.N.A.® Tissue DNA Kit (Omega Bio-tek) was used for DNA extraction, generating ~ 8 Kb (Kilobase) long fragments. Sequencing was carried out in schools using the Nanopore minION sequencer. An average of 1 μg per sequencer was loaded using FLO-MIN106D flow cells (R9). Sequencing time was 24 h using MinKNOW software, with an approximate throughput of 4 Gb (giga bases) obtained per sample.

#### b. De-novo assembly

For each species, base calling was performed using Guppy v4.2.2 software (Oxford Nanopore Technologies). For quality control, both LongQC v1.0 [76] and Nanoplot v1.33.1 software [77] were used, since they provide complementary metrics for the analysis. Porechop v0.2.4 software [78] was used for trimming of adapters. The sequence filtering step, according to phred quality scores, was performed with NanoFilt v2.7.1 software [77]. Three different filterings were performed based on minimum phred quality and minimum read length (minimum length 1000 bp and minimum quality 12, minimum length 1000 bp and minimum quality 10, minimum length 500 bp and quality 10), to later compare the quality of the generated assemblies. Flye v2.8.1 software [79] was used to generate the 3 assemblies per species. Once the preliminary assemblies were obtained, quality analysis of the assemblies was performed using traditional metrics (N50, number of fragments) and by searching for highly conserved core insect genes using the BUSCO pipeline [60].

Subsequently, the polishing step was performed with Medaka v1.2.0 software [80], generating final assemblies. Finally, the assemblies were compared using the metrics previously mentioned and a consensus assembly for each species was then used in subsequent analyses.

#### c. Structural and functional annotation

Annotation of transposable elements, tandem repeats and low complexity sequences was performed with RepeatModeler v2.0.1 [49] and RepeatMasker v4.1.1 [50]. tRNAscan-SE [52] was used for tRNA annotation. Ribosomal RNAs, lncRNAs, miRNAs, snRNAs and snoRNAs were annotated using the Infernal v1.1.2 software [81] with the Rfam 14.6 database [51].

For coding sequence structural annotation, the BRAKER2 v2.1.5 pipeline [53] was used, which uses two online software programs to perform its gene predictions: GeneMark-ET and AUGUSTUS. Both tools make use of transcriptomic data to perform training models for coding sequence prediction (CDS). The transcriptomic data used for both species were obtained from the following sources:

- The RNA-Seq data of *Euborellia annulipes* corresponds to samples obtained as a part of previous research by one of us (P.I.; unpublished results).
- The *Forficula auricularia* RNA-Seq results correspond to data obtained by Roulin and collaborators [82]. Samples were accessed through the NCBI Sequence Read Archive, with the following accession numbers SRR1043671, SRR1048074, SRR1051467.

RNAseq data was analyzed with FastQC v0.11.9 [83] and Multiqc v1.10.1 [84]. Quality trimmings were performed with Trimmomatic v0.39 software [85], and subsequently aligned to their respective genomes with the STAR v2.7.8a software [86] in order to be used in the BRAKER2 pipeline.

Functional annotation was performed using the BLAST v2.11.0 tool [87] against the SwissProt databases [54] and a "custom" insect database generated from all insect protein sequences present in NCBI accessed on May 12, 2021.

Orthologous groups were annotated using eggNOG-mapper v2 software [68] with eggNOG v5.0 database [55], which also provided annotation in Gene Ontology terms.

#### d. Protein orthogroup relationships

To compare the proteome of sequenced earwig species and the proteome of other insects, we decided to incorporate the protein sets available in NCBI of 8 species belonging to the winged insect group Pterygota (Table 8) and carried out an orthogroup analysis. For this end we used Orthofinder v2.5.2 software [67], which provides information about inter-species orthogroups, species specific orthogroups, orthologs and duplication events.

**Table 8:** Accession number of species included in orthogroup analysis

Species	Accession number
*Anisolabis maritima*	GCA_010014785.1
*Aedes aegypti*	GCA_000004015.3
*Anopheles sinensis*	GCA_000441895.2
*Blattella germanica*	GCA_003018175.1
*Coptotermes formosanus*	GCA_013340265.1
*Cryptotermes secundus*	GCA_002891405.2
*Ephemera danica*	GCA_000507165.2
*Zootermopsis nevadensis*	GCA_000696155.1

#### e. Enrichment of GO terms

Using both the EggNOG and Orthofinder outputs, a GO term enrichment analysis was performed using the Ontologizer v2.0 tool [88] to analyze species-enriched biological processes based on the gene subgroups of interest: genes belonging to species-specific orthogroups of both *E. annulipes* and *F. auricularia*. Enrichment was performed taking as the universe all GO terms annotated in the genomes of each species and as a subgroup the GO terms belonging to orthogroups unique to both *Forficula auricularia* and *Euborellia annulipes*. Enrichment was performed using the "Parent Child" method with Bonferroni multiple testing correction, taking as significant those GO terms with an adjusted p-value of less than 0.01. These results were further processed through the Revigo tool [56], that allows summarizing and visualizing long lists of GO terms by finding subgroups of related terms, choosing a representative of such subgroup guided by the statistical value previously inferred by Ontologizer.

## Supplementary Information

**Additional file 1.** List of enriched GO terms corresponding to genes present in orthogroups exclusive to *E. annulipes.*

**Additional file 2.** List of enriched GO terms corresponding to genes present in orthogroups exclusive to *F. auricularia.*

**Additional file 3.** Flier of the school competition for participation in the sequencing activity.

**Additional file 4.** Structural annotation of protein coding genes for both earwig species.

## Data Availability

The datasets generated during the current study are available in the NCBI repository, under the accession numbers PRJNA792355 and PRJNA792391.

## Abbreviations

bp: Base pairs
Gb: Giga base
FA: *Forficula auricularia*
EA: *Euborellia annulipes*
ncRNA: Non-coding RNA
CDS: Coding sequence
GO: Gene ontology
