# Supplementary material for: DNA sequencing in the classroom: complete genome sequence of two earwig (Dermaptera; Insecta) species
Source: Biol Res. 2023 Feb 17;56:6. doi: 10.1186/s40659-023-00414-9 (PMC9935246; doi:10.1186/s40659-023-00414-9)
Supplement: Supplementary file 3 — Additional file 3. Flier of the school competition for participation in the sequencing activity. [file 40659_2023_414_MOESM3_ESM.pdf]

SEGUNDO CONCURSO ESCOLAR:

CHILE SECUENCIA A CHILE
(PROYECTO 1000 GENOMAS)

Postulaciones hasta el 1 de julio 2019

En el marco de la iniciativa 1000 Genomas Chile, los centros científicos de excelencia que lideran el proyecto llaman a la comunidad escolar del país a participar directamente a través de este concurso. Invitamos a l@s alumn@s, en conjunto con sus profesor@s del área científica, a sumarse a este espacio de exploración y ser parte de un experimento comunitario y nacional en que secuenciaremos el genoma de un organismo, resultado que se incorporará al proyecto 1000 Genomas. El objetivo de este concurso es acercar los conceptos y alcances de la genómica a la ciudadanía y en especial a los niños y niñas interesad@s en ciencias. El experimento se llevará a cabo durante el segundo semestre de 2019 en los liceos o colegios seleccionados o en los laboratorios de los Centros de Excelencia que patrocinan el evento. A los lugares seleccionados viajarán científicos de la iniciativa y se compartirán y compararán los datos obtenidos por todos los participantes. Previamente, existirá un periodo de preparación teórica y práctica y se entregará un instructivo que detalla el protocolo experimental a seguir. También se proveerán todos los materiales requeridos, incluyendo computadores con el software apropiado y los equipos de secuenciación, sin costo.

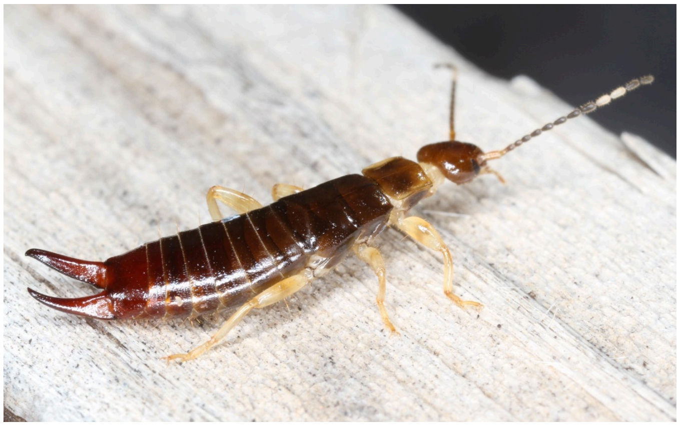

Al final del experimento, se recopilará la información y se entregarán los resultados obtenidos por todos desde el Centro de Regulación del Genoma (CRG). En esta ocasión, proponemos analizar un tipo de organismo que se encuentra presente en todo el territorio: un insecto que pertenece al órden Dermaptera, comúnmente conocido como "tijereta". El resultado será original (no se ha secuenciado previamente el genoma de especies de este tipo hasta ahora) y los datos obtenidos serán publicados y depositados en bases de datos de acceso público. La recolección del organismo a secuenciar (un sólo individuo) deberá realizarse usando cuidadosos registros de ubicación y documentación con imágenes de la más alta calidad posible. Para garantizar el adecuado respeto a las consideraciones bioéticas, la manipulación de los animales será hecha por personas autorizadas expresamente para ello.

cgr Center for Genome Regulation · ACCDiS Advanced Center for Chronic Diseases · CMM Center for Mathematical Modeling · Gero · iBio · Oxford Nanopore Technologies

CONICYT Ministerio de Educación, Gobierno de Chile · explora Un Programa CONICYT · Ministerio de Economía, Fomento y Turismo, Gobierno de Chile

SEGUNDO CONCURSO ESCOLAR:

CHILE SECUENCIA A CHILE

(PROYECTO 1000 GENOMAS)

Postulaciones hasta el 1 de julio 2019

¿Quienes pueden participar?

Cada grupo que postula consiste de entre 3 y 10 escolares de liceos municipales o particulares subvencionados en cualquier parte del territorio de Chile. Deben estar bajo la supervisión de un profesor o profesora responsable, idealmente del área científica. El grupo que postula debe contar con la autorización del director del establecimiento educacional; puede postular un sólo grupo por establecimiento. No se requiere ningún material para participar; sólo la dedicación de tiempo necesaria para llevar a cabo el experimento (algunas horas para la preparación y dos días durante el evento). Los participantes deben estar de acuerdo en que sean filmados y fotografiados para las actividades de difusión del proyecto, así como de aparecer por *video streaming* que puede ser transmitido públicamente. Para esto, deben contar con el consentimiento informado por escrito de su apoderad@.

¿Cuando ocurrirá?

Se puede postular hasta el 1 de julio de 2019. Una vez seleccionados los grupos participantes, se les entregarán oportunamente las instrucciones y el material. Se intentará que cada grupo cuente con la visita previa de un científico de apoyo para orientar el trabajo. En coordinación con los grupos seleccionados, se acordará una fecha para la realización del evento de secuenciación, el cuál ocurrirá durante el segundo semestre (agosto-noviembre) de 2019.

Bases y Formulario:

www.1000genomas.cl/concurso2019

cgr Center for Genome Regulation

CMM Center for Mathematical Modeling

ACCDiS ADVANCED CENTER FOR CHRONIC DISEASES

Gero

iBio Instituto Milenio de Biología Integrativa

CONICYT Ministerio de Educación Gobierno de Chile

explora Un Programa CONICYT

Ministerio de Economía, Fomento y Turismo Gobierno de Chile

Oxford NANOPORE Technologies
