# Supplementary material for: DNA sequencing in the classroom: complete genome sequence of two earwig (Dermaptera; Insecta) species
Source: Biol Res. 2023 Feb 17;56:6. doi: 10.1186/s40659-023-00414-9 (PMC9935246; doi:10.1186/s40659-023-00414-9)
Supplement: Supplementary file 4 — Additional file 4. Structural annotation of protein coding genes for both earwig species. [file 40659_2023_414_MOESM4_ESM.pdf]

Additional File 4. Structural annotation of protein coding genes for both earwig species.

	Forficula auricularia	***Euborellia annulipes***
Number of genes	31779	40028
Number of CDSs	33587	42675
Number of exons	147010	198427
Number of introns	117244	162278
Number of genes overlapping	156	119
Number of single exon gene	10026	11177
Total gene length	310662.7 kb	250644 kb
Total mRNA length	361038 kb	301001.8 kb
Total CDSs length	30234.7 kb	44792.7 kb
Total exon length	43,150 kb	59108 kb
Total intron length	330803.6 kb	256209 kb
Total 5' UTR length	7758 kb	9722 kb
Total 3' UTR length	5157 kb	4593 kb
Mean gene length	9.8 kb	6,3 kb
Mean CDSs length	0.9 kb	1.049 kb
Mean exon length	293 bp	297 bp
Mean intron length	2821 bp	1578 bp
Mean 5' UTR length	3454 bp	2351 bp
Mean 3' UTR length	3274 bp	1921 bp
Mean mRNAs per gene	1.1	1.1
Mean CDSs per mRNA	1.0	1.0
Mean exons per mRNA	4.4	4.6
Mean introns per mRNA	3.5	3.8
Longest gene	266.4 kb	228.5 kb
Longest CDS	42654 bp	50282 bp
Longest exon	25019 bp	14656 bp
Longest intron	146895 bp	117750 bp
Shortest gene	50 bp	92 bp
Shortest CDS	17 bp	9 bp
Shortest exon	3 bp	3 bp
Shortest intron	7 bp	6 bp
